# Understanding patient pathways and care delays in India: implications for early leprosy detection

**DOI:** 10.5588/pha.26.0037

**Published:** 2026-08-24

**Authors:** S. Das, G. Ramaswamy, D. Konda, D. Nair, J.P. Tripathy, M. Vijayageetha, K. Manoha, P. Roy, G. Gaffoor, S. George, N. Agarwal

**Affiliations:** 1 All India Institute of Medical Sciences, Bibinagar, Telangana, India;; 2 Independent Researcher, Scottsdale, AZ, USA;; 3 All India Institute of Medical Sciences, Nagpur, Maharashtra, India;; 4 Tufts University of School of Medicine, Boston, MA, USA.

**Keywords:** diagnostic delay, health-seeking behaviour, patient care pathways, mixed-methods study, SORT-IT, operational research

## Abstract

**SETTINGS:**

Dermatology clinic in a public tertiary hospital in Telangana, India.

**OBJECTIVES:**

Among newly diagnosed leprosy patients (2023–2025), to describe patient care pathways, quantify patient and health system delays and compare across private and public sectors, and explore patient and provider perceptions about care delays.

**DESIGN:**

Mixed-methods study with cross-sectional quantitative (questionnaire-based interviews) and descriptive qualitative components (in-depth interviews).

**RESULTS:**

Of 90 eligible, 46 (51%) were recruited. The median (inter-quartile range [IQR]) number of providers consulted was 3 (2–3). Three fourths first sought care from a public provider. Median (IQR) overall delay was 108 days (40–255), patient delay of 73 days (16–158) (symptom onset to first health care contact) and health system delay of 14.5 days (11–39) (first health care contact to treatment initiation). Patients initially approaching private sector had longer median health system delay than those seeking public sector (28 vs. 13 days, *P* = 0.02). Poor symptom awareness, symptom neglect, and stigma contributed to patient delay, while poor provider capacity and long-distance travel contributed to health system delays.

**CONCLUSION:**

Delays in leprosy care are largely driven by patient delay and missed diagnostic opportunities, particularly in the private sector. Strengthening community awareness and private sector engagement is essential for early detection.

## Graphical abstract

**Figure:**
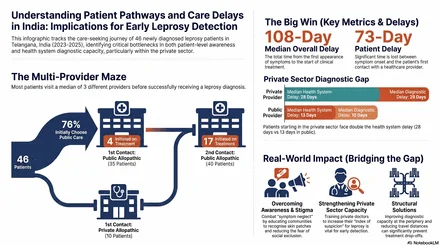

The WHO Neglected Tropical Diseases Roadmap 2021–2030 envisions a world with ‘zero leprosy’ by 2030, defined as zero infection and disease, zero disability, and zero stigma and discrimination.^1^ Achieving this goal requires interruption of transmission through early case detection and prompt initiation of treatment. Delays in diagnosis undermine these efforts by allowing continued transmission and increasing the risk of irreversible nerve damage and disability.^2^ Aligned with the global roadmap, India’s National Strategic Plan (NSP) and Roadmap for Leprosy 2023–2027 prioritises acceleration of case detection as a key strategic pillar. The NSP explicitly identifies delayed diagnosis as a major programmatic challenge.^3^ These delays occur at two levels: patient delay (time between onset of symptoms and first health care contact) and health system delay (time between first health care contact and treatment initiation).^4^ Two studies from India have reported substantial median delays in care seeking and diagnosis, ranging between 4–8 and 2–18 months, respectively.^2,4^ A mixed-methods study from India has highlighted poor patient awareness of symptoms, stigma, initial consultation with private providers unfamiliar with leprosy management, and reduced capacity of health care providers (HCPs) in leprosy control as the key reasons for delayed diagnosis.^4^ Studies from countries such as China, Nepal, Brazil, Ethiopia, and Paraguay have similarly reported substantial patient and health system delays in leprosy care, echoing the findings observed in India.^5–10^

While these studies report the magnitude and determinants of delay, a critical gap remains in understanding the pathways of care that patients navigate to access care. A recent systematic review emphasises that misdiagnosis and multiple consultations with inappropriate providers before correct diagnosis are the key contributors to delayed diagnosis of leprosy.^11^ However, there is a paucity of studies that systematically document the number, type, and sequence of care providers approached by patients, particularly in the Indian context. One study conducted in 2000 by Nicholls et al. in West Bengal reported that the number of help-seeking actions before diagnosis of leprosy ranged from 1 to 7.^12^ Understanding these patient care pathways is essential for identifying missed opportunities for early detection, inappropriate care seeking, and referral patterns. Such insights are directly relevant to the NSP’s call for stronger engagement with private providers, traditional healers, and other non-governmental stakeholders to improve early diagnosis and treatment initiation.^3^

Tertiary care hospitals act as higher referral centres where patients arrive (both referred and self-referred) after visiting multiple providers, allowing detailed reconstruction of their care pathways and identification of delays, misdiagnoses, and referral gaps. Against this background, we designed and implemented a mixed-methods study among the newly diagnosed leprosy patients registered for care at a tertiary hospital in a moderately endemic district of Telangana, India, during 2023–2025, to i) determine the number, type, and sequence of care providers approached by patients prior to diagnosis, ii) determine the magnitude of patient and health system delays and compare these delays across patients seeking care in the public and private sector at the first contact, and iii) explore the patients’ and providers’ perceptions regarding patient and health system delays. While the quantitative component maps the sequence of providers and measure delays, the qualitative component helps us explore underlying reasons. Together, they provide a more comprehensive and actionable understanding of delays in leprosy care, justifying the use of a mixed-methods design.

## METHODS

A mixed-methods study with a quantitative component (cross-sectional structured interviews with patients) and a descriptive qualitative component (in-depth interviews [IDIs] with patients and HCPs).

### Study setting

The National Leprosy Eradication Programme is a centrally sponsored scheme providing free-of-cost services for prevention, diagnosis, treatment, and rehabilitation of leprosy patients diagnosed either passively or through active case-finding efforts at all public health facilities across India.^13^ Apart from public health facilities, patients also seek care from private health institutions and traditional healers.

### Specific setting

The present study was conducted in the Dermatology Outpatient Department (OPD) of AIIMS Bibinagar which is a tertiary care institute in Telangana serving ∼50,000 patients monthly, largely from rural and peri-urban areas of Yadadri Bhuvanagiri district, a moderately endemic district for leprosy.^14^ The OPD has a monthly footfall of ∼3,000 patients. Individuals suspected of leprosy based on clinical assessment undergo confirmatory tests such as slit skin smear microscopy and skin biopsy. Details of confirmed cases are recorded in a separate register; then they are counselled and referred to the District Hospital for treatment initiation, contact screening, and chemoprophylaxis. They are advised monthly follow-up at the OPD to monitor treatment response.

### Study population

All newly diagnosed leprosy patients registered at the Dermatology OPD between January 2023 and December 2025 constituted the study population. Besides, the HCPs involved in management of leprosy patients in the hospital also formed the study population for the qualitative component.

### Sample size and sampling

All the eligible leprosy patients in the study period as mentioned above were consecutively recruited into the study. For the qualitative component, purposive sampling (maximum variation) was done among the patients to represent patients with varied clinical spectrum. A total of 10 patients (eight with no deformity or grade 1 deformity and two with grade 2 deformity) including a mix of patients who approached a public or a private provider as the first contact and 3 HCPs (two resident doctors and a consultant dermatologist) were selected. To minimise some recall bias, patients were selected from those diagnosed in 2024 and 2025.

### Data collection

A line list of eligible patients with contact details was obtained from registers and they were contacted. Patients were interviewed during Dermatology OPD visits or via telephone. A structured questionnaire collected data on care pathways, patient and health system delays, and socio-demographic, clinical, and behavioural factors. Information on providers visited and visit dates was used to map care pathways, while dates of symptom onset, diagnosis, and treatment initiation were used to assess delays. Providers included informal (traditional healers) and formal (licensed public or private, including non-allopathic) practitioners. To reduce recall bias, data were cross-verified with treatment records where available, aided by the traditional Indian calendar. When exact dates were unknown, the first day of the reported month was used. IDIs with patients and HCPs were conducted in a private room in the Dermatology department by trained investigators (SD and KM), who were trained in qualitative research (degree equivalent to Masters in Public Health) using pilot-tested guides with probes. Interviews were held at convenient times in local language (Telugu) or English, with only participant and investigators present. Each interview lasted ∼15–20 min, was audio-recorded, and supplemented with field notes.

### Data entry and analysis

Quantitative data were captured in Epicollect5 (V87.2.1, Centre for Genomic Pathogen Surveillance, University of Oxford, 2025), a mobile based data capture tool, and analysed using Jamovi software (V2.7.4.0, The Jamovi Project, Sydney, Australia, 2025). Categorical and continuous variables were compared across groups using χ² and *t* test, respectively. The number of providers approached and patient and health system delays are summarised using median and inter-quartile range (IQR) and compared across groups by the Mann–Whitney test. Pathways to care have been described using a Sankey diagram with the thickness of the lines corresponding to the number of patients seeking care. The delays are defined as follows: 1) patient delay (symptom onset to first visit to a care provider); 2) health system delay (first visit to a care provider to treatment initiation), which is further divided into: diagnostic delay (first visit to a care provider to diagnosis) and treatment delay (diagnosis to treatment initiation); and 3) total delay (symptom onset to treatment initiation).

For qualitative data, IDI transcripts were prepared in English on the day of interview using notes and audio recordings by SD. Thematic analysis was conducted by SD using ATLAS.ti (V9.25.1, ATLAS.ti Scientific Software Development GmbH, Berlin, Germany, 2026) following standard procedures.^15^ Both deductive and inductive coding were used, and similar codes were grouped into themes to develop a thematic framework. A second qualitative researcher (VG) reviewed coding and theme development, with discrepancies resolved through discussion. The quantitative and qualitative findings are reported according to ‘Strengthening the Reporting of Observational Studies in Epidemiology (STROBE)’ and ‘Consolidated Criteria for Reporting Qualitative Research (COREQ)’ guidelines, respectively.^16,17^

### Ethical statement

Ethics approval was obtained by the Institutional Ethics Committee of AIIMS Bibinagar (AIIMS/BBN/IEC/Oct/2025/830-R). Informed written consent was taken from the study participants and recorded verbal consent was taken prior to telephonic interviews.

## RESULTS

Of 98 newly diagnosed leprosy patients registered during the study period, 8 were duplicate entries. Of remaining 90, 24 were non-responders, 10 had wrong contact details, 9 denied diagnosis of leprosy, and 1 died due to unknown cause, resulting in final inclusion of 46 (51%) patients. Included and excluded patients did not differ significantly by age, residence, or type of leprosy, except gender. Detailed results are available at https://doi.org/10.6084/m9.figshare.31883263.

### Patient characteristics

Table 1 summarises the socio-demographic and clinical characteristics of the participants. Majority were males (41, 89.1%), married (32, 69.6%), with a median age of 39.6 years. Nearly half came from urban areas (25, 54.3%) and a similar number from the neighbouring districts. Most had grade 0 or 1 deformity (41, 89.1%) with five (10.9%) having grade 2 deformity. About three fourths reported skin patch with loss of sensation as the first reported symptom.

**TABLE 1.** Socio-demographic and clinical characteristics of the newly diagnosed leprosy patients registered for care at a tertiary hospital in Telangana, during 2023–2025.

Characteristics	N (%)
Total	46 (100)
Age in years (mean, SD)	39.6 (13.0)
Sex	
Male	41 (89.1)
Female	05 (10.9)
Residence	
Urban	25 (54.3)
Rural	21 (45.7)
Locality	
Within the district	16 (34.8)
Neighbouring districts	25 (54.3)
Neighbouring states	05 (10.9)
Marital status	
Married	32 (69.6)
Single/separated/widowed	14 (30.4)
Education (years of schooling) median (IQR)	10 (0–12)
Socio-economic class **^A^**	
Upper	28 (60.8)
Middle/lower	18 (39.2)
Distance to nearest public health facility	
5 km and above	24 (52.2)
Less than 5 km	22 (47.8)
History of leprosy among the household members	
Yes	06 (13.0)
No	40 (87.0)
Grade of deformity at diagnosis	
Grade 0	08 (17.4)
Grade 1	33 (71.7)
Grade 2	05 (10.9)
Type of leprosy (WHO)	
Paucibacillary	24 (52.2)
Multibacillary	19 (41.3)
Missing	03 (6.5)
Current status	
On treatment	16 (34.8)
Treatment completed	27 (58.7)
Defaulted	03 (0.07)

SD = standard deviation; IQR = inter-quartile range.

A Modified BG Prasad Classification.

### Patient care pathways

The median (IQR) number of HCPs approached by the patients was 3 (2–3). The Figure describes the pathways of the patients’ seeking health care for leprosy. At the first point of contact, three fourths (35, 76.1%) sought care from a public allopathic provider, of whom, 4 were initiated on treatment, 10 (21.7%) visited a private allopathic provider, and 1 approached an informal provider. Of 42 patients who consulted a second provider, 40 visited public allopathic providers, and 17 were initiated on treatment. All those who visited a private provider (n = 10) or an informal provider (n = 1) during their first consultation approached a public allopathic provider during their second consultation. Twenty-five patients proceeded to consult a third HCP, and six patients consulted a fourth HCP.

**FIGURE.**
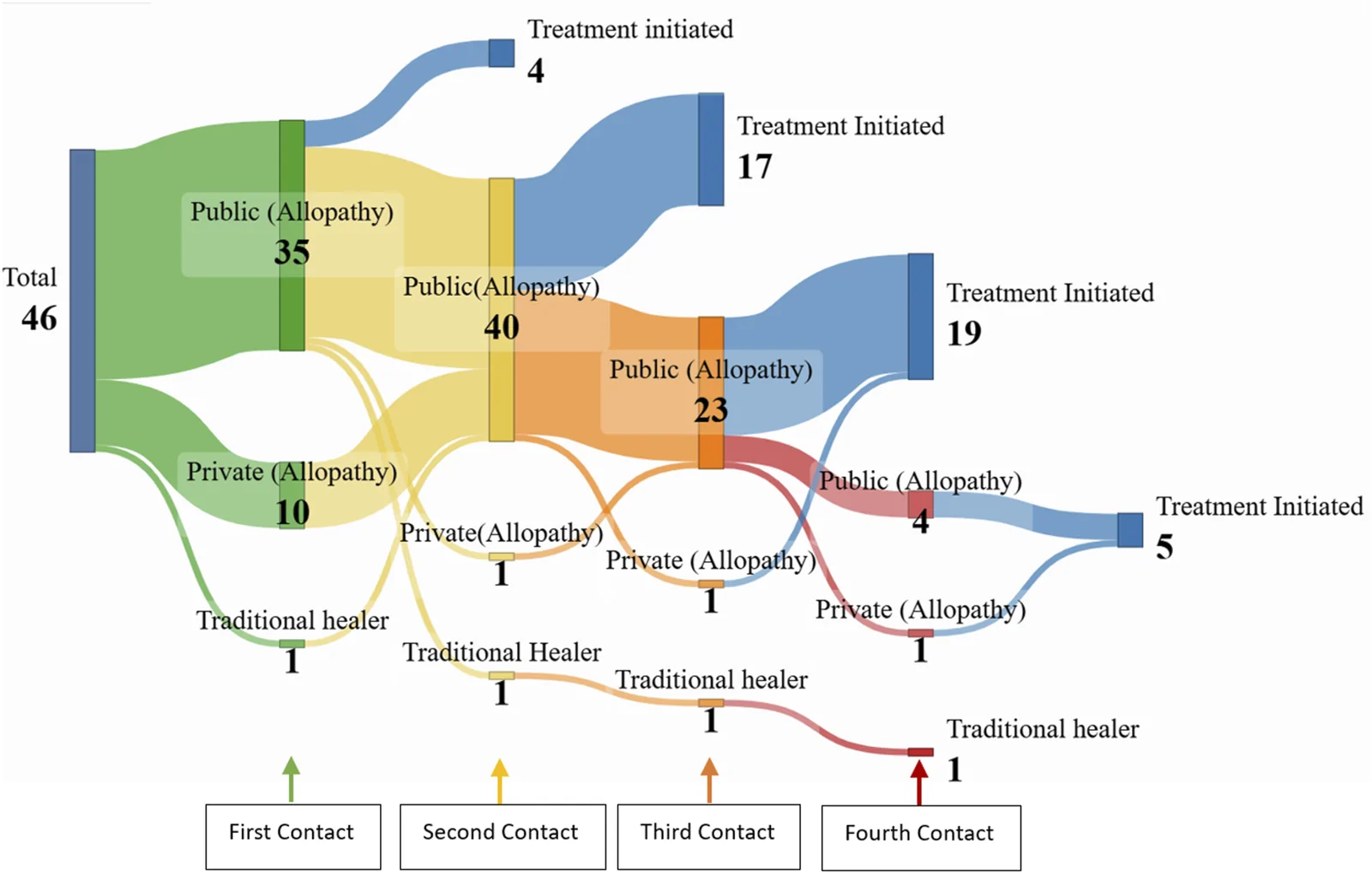
Pathways to care undertaken by the newly diagnosed leprosy patients registered for care at a tertiary hospital in Telangana, during 2023–2025 (N = 46).

### Delays in care

As described in Table 2, the median (IQR) overall delay was 108 days (40–255). Overall median (IQR) patient and health system delays were 73 days (16–158) and 14.5 days (11–39), respectively. Further, diagnostic and treatment delays were 12 days (9–30) and 1.5 days (1–3), respectively. The median (IQR) patient delay among patients who first approached an allopathic public provider was 89 days (30–158), compared to 21 days (1.5–193) among those who initially consulted an allopathic private provider, although this difference was not statistically significant. However, patients initially seeking care at an allopathic private provider experienced a longer health system delay of 28 days (16–122) compared to 13 days (10–27.5) in the other group (*P* value = 0.01). This delay was mainly contributed by diagnostic delay (*P* value = 0.02).

**TABLE 2.** Patient delays and health system delays among the newly diagnosed leprosy patients registered for care at a tertiary hospital in Telangana, 2023–2025 (N = 46).

	Delay in median number days (IQR)			
Type of delay	Overall	Public health care provider at first contact	Private health care provider at first contact	*P* value
Patient delay (symptom onset to first visit to a care provider)	73 (16–158)	89 (30–158)	21 (1.5–193)	0.26
Health system delay (first visit to a care provider to treatment initiation)	14.5 (11–39)	13 (10–27.5)	28 (16–122)	0.02*
Diagnostic delay (first visit to a care provider to diagnosis)	12 (9–30)	10 (7–16.5)	28 (14–110)	0.02*
Treatment delay (diagnosis to treatment initiation)	1.5 (1–3)	1 (1–3)	2 (0.5–3.5)	0.90
Total delay (symptom onset to treatment initiation)	108 (40–255)	104 (48–211)	214 (68–266)	0.51

*P value statistically significant; estimates presented indicate delays measured in terms of median number of days (IQR).

IQR = inter-quartile range.

Two major themes were explored: 1) factors contributing to delays and 2) factors reducing delays. Both patient and health system level factors were investigated (Tables 3 and 4).

**TABLE 3.** Factors contributing to patient and health system–level delays in leprosy care as perceived by patients and health care providers at a tertiary hospital in Telangana, India, 2023–2025.

Themes	Sub-themes	Categories	Codes	Verbatims
Factors contributing to patient and health system delays	Patient-level delays	Lack of knowledge	Lack of awareness of symptoms	I thought it (skin patch) was because of winter. It came at that same time. – 45-year-old first approaching a public provider
				I delayed because I thought it (skin patch) would go away. I was also busy with work. – A 39-year-old first approaching a public provider
				Patients may not recognise early symptoms as significant. – HCP 1
		Neglect of symptoms	Intention to seek care only when symptoms worsen	Patients tend to neglect symptoms initially and seek care only when the condition worsens and interferes with daily activities. – HCP 1
			Late presentation	Many patients still present late. They often come after several months of symptoms. – HCP 2
		Stigma	Marginalisation and social/self-isolation	I am not telling anyone outside. At home we stay together. If I go out for work, I keep some distance. – 20-year-old first approaching a public care provider
				Some outsiders do not come near them (leprosy patients) or like to touch them. – 27-year-old first approaching a private care provider
				There is also significant fear of stigma, especially among married individuals. A few (married female patients) have been sent back to their parents' homes or abandoned. – HCP 3
				Even after 8 months of treatment. My symptoms improved, and the patch became lighter. The numbness reduced. But still, they kept my utensils separate and asked me to sleep separately. – A 33-year-old first approaching a public provider
				But at my in-laws’ place, they treated me like I was contagious. – A 39-year-old first approaching a public provider
			Fear of infecting family	My mother cried. They (family) asked if it would spread to them. – A 33-year-old first approaching a public provider
			Self-isolation	I understood they (family members) were scared, but it felt like I was different. Sometimes I avoided sitting with them even if they didn’t say anything. – A 33-year-old first approaching a public provider
		Distance to facility and travel	Financial burden	Yes, I felt the money was getting exhausted. – A 17-year-old first approaching a private provider
	Health system–level delays	Untrained care provider	Lack of access to qualified care provider	They often consult unqualified practitioners or RMPs, who provide reassurance that the condition is minor. – HCP 2
				Local practitioners may not suspect leprosy. – HCP 3
			Informal care providers	False assurance (by the informal health care provider) leads to delay. – HCP 2
			Limited awareness among care providers	Some providers may initially give alternative diagnoses due to limited awareness. – HCP 1
		Incorrect diagnosis	Incorrect diagnosis among the qualified providers	A biopsy at one hospital came normal. Later, at …, they did another biopsy. It did not confirm leprosy fully, but it showed something small and slight loss of sensation. – 27-year-old first approaching a private care provider
		Lack of guidance	Lack of proper guidance	But no one clearly explained how long to take them, where exactly to get them regularly, or what the next steps were. – A 27-year-old first approaching a public provider
		Travel	Distance travelled for treatment	It (travelling) was a struggle. It is about 40–50 km. – A 17-year-old first approaching a private provider
				The only problem was traveling every month. It is far from our village. – A 20-year-old first approaching a public provider
				After referral, continuity of care may be affected by travel distance. – HCP 3
		Unavailability of treatment	Unavailability of treatment at the diagnosing facility	They are referred to the nearest PHC for multidrug therapy. We do not provide the tablets here. – HCP 1
		Improper follow-up	Irregular follow-up	Some patients come for regular follow-up, but many do not. – HCP 1
			No felt need of follow-up	However, since medicines are provided at PHCs, they usually return (to the tertiary facility) only when problems worsen. – HCP 2

HCP = health care provider; RMP = rural medical practitioner; PHC = primary healthcare centre.

**TABLE 4.** Factors reducing patient and health system–level delays in leprosy care as perceived by patients and health care providers at a tertiary hospital in Telangana, India, 2023–2025.

Factors reducing patient and health system delays			
Patient-related factors	Proximity to facility	Less distance	I came (to the tertiary centre) because it is available nearby. Regarding distance, I am in Bhongir. I am nearby. There is no trouble. – 47-year-old first approaching a public provider
	Patient awareness	Knowledge about leprosy	Yes. I had heard about it (leprosy) before… from school and also from people in the village. – A 33-year-old first approaching a public provider
			I found out after diagnosis. But I knew that such a disease existed. – A 17-year-old first approaching a private provider
Health system–related factors	Provider awareness	Referral to higher facility	After diagnosis, we refer patients to their local PHC for multidrug therapy. – HCP 2
			I went to Ghatkesar Government Hospital. They examined me and referred me here (tertiary facility) for further management. – A 39-year-old first approaching a public provider
		Professional counselling	The doctor said there was no need to maintain distance. I stayed with my family. – 45-year-old first approaching a public provider
			They (provider) told me it is curable, how long I need to take tablets, possible side effects, and what to do if reactions happen. – A 27-year-old first approaching a public provider
			We routinely counsel patients that leprosy is not highly contagious and that it is safe to interact normally. – HCP 2
	Programmatic management	Active case finding	Government health workers came door-to-door and asked me to get it (skin patch) checked again. – 27-year-old first approaching a private care provider
		Medicines at periphery	No trouble. They (provider) give medicines every month. – 20-year-old first approaching a public care provider
			Medicines were available every month. That was not a problem. – A 33-year-old first approaching a public provider
	Family and societal support	Community acceptance	I went to Shivananda (leprosy hospital) and saw patients there. Everyone was living together well. There was nothing like that. – 47-year-old first approaching a public provider
		Acceptance by family	My relatives looked after me well. – 45-year-old first approaching a public provider
			My family was very supportive. From the beginning, they said it is just a disease and it will be treated. – A 27-year-old first approaching a public provider

HCP = health care provider.

### Patient level factors contributing to delays

Lack of awareness and neglect of symptoms: Many patients did not recognise initial symptoms, such as skin patches, as indicative of disease and often attributed them to benign or seasonal causes. As one participant noted:

I thought it (skin patch) was because of winter. It came at that same time.

HCPs similarly emphasised that early symptoms are frequently overlooked by patients. This lack of awareness was compounded by a tendency to neglect symptoms, with patients delaying care until the condition worsened or began to interfere with daily activities. As described by a provider:

They tend to neglect symptoms initially and seek care only when the condition worsens.

Stigma: Participants described experiences and fears of marginalisation and social exclusion, which discouraged them from seeking timely care:

I was more worried about how others would react than about the disease itself.

Both patients and HCPs reported instances of exclusion within family and community settings. A provider highlighted that:

A few (married female patients) have been sent back to their parents’ homes or abandoned.

Experiences of isolation within households were also evident:

Even after eight months of treatment… they kept my utensils separate and asked me to sleep separately.

Fear of transmitting the disease to family members further contributed to anxiety and delay:

My mother cried. They (family) asked if it would spread to them.

### Health system factors contributing to delays

Both patients and HCPs highlighted the role of non-qualified and informal practitioners, who provided false reassurances, thereby delaying appropriate care. As noted by providers:

They often consult unqualified practitioners or RMPs, who provide reassurance that the condition is minor.

Even among qualified providers, limited awareness and low index of suspicion resulted in misdiagnosis or delayed diagnosis.

Structural barriers such as travel distance also affected timely access and continuity of care, with patients describing the burden of repeated long-distance travel.

‘It was a struggle… about 40–50 kilometres’ and ‘the only problem was traveling every month’.

### Factors reducing delays

Proximity to health facilities and prior awareness of leprosy facilitated early care seeking, while provider-initiated referrals, effective counselling and guidance, and active case finding supported early diagnosis and treatment. In addition, consistent drug availability and strong family and social support helped improve treatment access and adherence.

## DISCUSSION

This is the first study from India providing a comprehensive patient care pathway analysis in leprosy from symptom onset until the initiation of treatment, integrating quantitative estimates and qualitative insights. We observed a substantial overall median delay, with patient delay being the major contributor. Patients approached three different care providers and about three fourths first sought care at a public health facility. Patients who initially sought care from private providers experienced significantly longer health system delays, primarily driven by diagnostic delay. The qualitative findings complement these results by highlighting poor symptom awareness and stigma as patient delays and improper diagnosis by less qualified, informal providers and poor physical access as key drivers of health system delay.

The overall delay observed in this study is consistent with prior evidence from India which reported a median of 6–18 months among patients with or without disability.^2,4,18^ Evidence from studies across multiple countries such as China, Ethiopia, Nepal, Indonesia, and Brazil demonstrates this long delay consistently.^5–9^ Although the absolute duration in our study appears shorter (∼2.5 months), the persistence of delays remains a critical concern. This underscores the fact that despite programme efforts, early detection is still suboptimal.

Patient delay constituted the major component of total delay in our study, aligning with previous literature from several countries.^2,4,7–9,18^ Poor awareness of early symptoms, especially the perception that skin lesions are trivial or self-limiting, along with stigma and economic barriers has been consistently reported as a key reason for delayed care seeking in India, China, and Brazil.^7,8^ The study in Brazil reported that participants who suspected they had leprosy but feared community isolation were 10 times more likely to wait longer before consulting a doctor for their symptoms.^7,8^ Qualitative explorations from Brazil and Paraguay have also highlighted lack of awareness about symptoms and stigma as the key drivers of patient delay.^10,19^ These findings highlight the need for sustained community awareness initiatives and targeted health education to improve early recognition of symptoms and care-seeking behaviour and reduce disease-related stigma.

Patients who first approached private allopathy providers experienced longer health system delays, mainly driven by diagnostic delays, which is supported by previous evidence.^2,4^ A systematic review of health system–related factors also identified first consultation with a private provider as a major contributor to diagnostic delay due to misdiagnosis, multiple consultations, and poor capacity of providers.^11^ In our study, although most patients eventually transitioned to public sector care for diagnosis and treatment, the initial encounters in the private sector likely resulted in missed or delayed diagnosis due to provider’s limited familiarity with leprosy, or lack of integration with national programme guidelines, a finding corroborated by qualitative interviews with HCPs. The median of three providers consulted prior to treatment in our study reflects missed opportunities for early diagnosis across both public and private sectors. Misdiagnosis due to poor capacity of the HCPs has also been reported as the main reason for health system delay in other similar settings in Indonesia, Brazil, and China.^5,7,8^ This highlights the need for strengthening referral systems, improving diagnostic capacity at peripheral levels, and engaging non-specialist and non-public health providers. Amidst these, it is encouraging that delays, as well as the number of consultations were lower among patients who first approached a public provider, likely reflecting the availability of specialist care at the tertiary study setting where this study was conducted.

The major strength of this study is the use of mixed-methods design and the consistency between quantitative and qualitative findings, which strengthens the credibility of the results and supports the overall interpretation. The study detailed care pathways, including the number, type, and sequence of providers consulted, addressing an important gap in the literature. We also adhered to STROBE and COREQ guidelines to report the findings. The study has several limitations. First, the inclusion of patients from a single tertiary care centre may limit generalisability. Second, inclusion of half of the eligible patients might introduce selection bias, given gender differences between included and non-included participants. Third, delays were based on patient recall and are subject to recall limitations. Fourth, the study includes only patients at a public facility, excluding those undiagnosed or treated in private or informal sectors. Fifth, the study was not specifically powered for subgroup comparisons; therefore, these findings should be interpreted accordingly. Finally, the qualitative component included a limited number of providers without any private sector representation, which may not fully reflect the diversity of health system perspectives.

These findings have important programmatic implications: i) campaigns to improve community awareness about early symptoms and reduce stigma and discrimination, ii) active case-finding strategies to promote early detection, iii) strengthening training and capacity of HCPs, especially in the private sector for early recognition and referral, and iv) strengthening private sector engagement through public–private mix models.

## CONCLUSION

Delays in leprosy diagnosis in this setting are substantial and largely driven by patient delay and accentuated by missed diagnostic opportunities, particularly in the private sector. Patients visit multiple providers before diagnosis, underscoring gaps in early recognition and referral. Strengthening community awareness and improving diagnostic capacity and engagement across both public and private sectors in treatment are critical to advancing toward ‘zero leprosy’.

## Supplementary Material




