# Supplementary material for: Understanding patient pathways and care delays in India: implications for early leprosy detection
Source: Public Health Action. 2026 Aug 24;16(3):237–43. doi: 10.5588/pha.26.0037 (PMC13634127; doi:10.5588/pha.26.0037)
Supplement: Supplementary file 1 [file pha26-0037_supplementarydata1.pdf]

Supplementary Table 1: Comparison of sociodemographic variables and type of leprosy between those included and those not included in the study

Characteristics	Included n(%)	Not Included n(%)	P value
Total	46 (100)	44 (100)	
Age in years* (mean, SD)	39.5, 13.2	42.9, 16.6	0.29
Sex			0.05
Male	41 (89.1)	32 (72.7)	
Female	5 (10.9)	12 (27.3)	
Residence			0.68
Urban	25 (54.3)	22 (50.0)	
Rural	21 (45.7)	22 (50.0)	
Locality			0.34
Within the district	16 (34.8)	16 (36.4)	
Neighbouring districts	25 (54.3)	26 (59.1)	
Other states	5 (10.9)	2 (4.6)	
Type of leprosy (n=87)			0.24
Paucibacillary	24 (55.8)	19 (43.2)	
Multibacillary	19 (44.2)	25 (56.8)	
Type of leprosy (Ridley-Jopling) (n=84)**			0.48
Tuberculoid (TT)	2 (5.0)	1 (2.3)	
Borderline Tuberculoid (BT)	16 (40.0)	16 (36.4)	
Borderline Lepromatous (BL)	3 (7.5)	2 (4.5)	
Lepromatous Leprosy (LL)	17 (42.5)	25 (56.8)	
Pure Neuritic (PN)	2 (5.0)	0 (0.0)	

*Student's t test, SD: Standard Deviation, **Fisher's exact test
